# Effectiveness of a systematic approach to promote intersectoral collaboration in comprehensive school health promotion-a multiple-case study using quantitative and qualitative data

**DOI:** 10.1186/s12889-015-1911-2

**Published:** 2015-07-05

**Authors:** Katharina K. Pucher, Math J. J. M. Candel, Anja Krumeich, Nicole M. W. M. Boot, Nanne K. De Vries

**Affiliations:** Department of Health Promotion, Maastricht University, Peter Debyeplein 1a, Box 616, 6200 MD Maastricht, Netherlands; Department of Methodology and Statistics, Maastricht University, Peter Debyeplein 1a, 6200 MD Maastricht, Netherlands; Department Department of Health, Ethics & Society, Maastricht University, Peter Debyeplein 1a, 6200 MD Maastricht, Netherlands; Department of Education and Research, Nieuw Eyckholt 300, 6419 DJ Heerlen, Netherlands

## Abstract

**Background:**

We report on the longitudinal quantitative and qualitative data resulting from a two-year trajectory (2008–2011) based on the *DI*agnosis of *S*ustainable *C*ollaboration (DISC) model. This trajectory aimed to support regional coordinators of comprehensive school health promotion (CSHP) in systematically developing change management and project management to establish intersectoral collaboration.

**Methods:**

Multilevel analyses of quantitative data on the determinants of collaborations according to the DISC model were done, with 90 respondents (response 57 %) at pretest and 69 respondents (52 %) at posttest. Nvivo analyses of the qualitative data collected during the trajectory included minutes of monthly/bimonthly personal/telephone interviews (*N* = 65) with regional coordinators, and documents they produced about their activities.

**Results:**

Quantitative data showed major improvements in change management and project management. There were also improvements in consensus development, commitment formation, formalization of the CSHP, and alignment of policies, although organizational problems within the collaboration increased. Content analyses of qualitative data identified five main management styles, including (1) facilitating active involvement of relevant parties; (2) informing collaborating parties; (3) controlling and (4) supporting their task accomplishment; and (5) coordinating the collaborative processes.

**Conclusions:**

We have contributed to the fundamental understanding of the development of intersectoral collaboration by combining qualitative and quantitative data. Our results support a systematic approach to intersectoral collaboration using the DISC model. They also suggest five main management styles to improve intersectoral collaboration in the initial stage. The outcomes are useful for health professionals involved in similar ventures.

## Background

Comprehensive school health promotion (CSHP) is endorsed by the WHO because of its broad perspective on children’s health, involving social and physical environments in and around school, community activity and redesigning health services. Moreover, it has been recognized as an effective means to improve children’s health and well-being [1–4].

The Dutch equivalent of CHSP is the Dutch Healthy School Approach (HSA). Basically, HSA targets demand-driven practices in school health promotion based on the epidemiological data of the school community, a prioritization of school needs, an assessment of important and modifiable determinants, the drafting and implementation of a multi-year school health plan, and its evaluation. All this is done jointly with multiple stakeholders who provide different expertise and fulfill different tasks [5–7]. At school level, the implementation of the HSA is professionally assisted by a ‘health promoting school advisor’, who represents various public services and providers (e.g. from the welfare, health, prevention and safety sectors) in individual contacts with schools. At local and regional levels, the public health services (PHSs) function as a linking pin (coordinator) between the education sector, health authorities and public services stakeholders (PSSs). Their coordinating role derives from a legal responsibility for the implementation of local public health policy and youth health care financed by the municipality [8].

While the popularity of the HSA and its adoption in Dutch regions is growing [9], its implementation remains complex [10, 11]. The establishment of a functional structure for the collaboration between the sectors involved poses important challenges in practice [12, 13]. The necessity of partnerships and networking and related challenges are not unique for the Dutch context, but are also acknowledged by studies in other countries in and outside Europe [14–17]. The increasing understanding of public health schools as social adaptive systems stimulates the study of intersectoral collaboration in CSHP. This perspective puts an emphasis on the need of joint work between relevant actors at different levels (individual, organizational, policy level) and from various sectors (health, education and authorities) to successfully implement CSHP [18, 19].

In the study at hand we investigated a systematic approach to the development of intersectoral collaborations based on a thorough analysis of facilitating and impeding factors and the use of appropriate change strategies. We studied the impact of this systematic approach quantitatively in a two-year trajectory supporting health professionals in its implementation. We also aimed to open up the ‘black box’ of collaborative processes, since collaboration research has found that these interactive processes are generally not very well understood [20, 21]. We therefore explored qualitatively if, and if so which, managerial activities were induced by the systematic approach. The benefits of a mixed methods approach (including qualitative and quantitative data) over qualitative or quantitative research alone have been acknowledged in inquiries evaluating health promotion in schools [22] and studying collaboration in public health [23]. In our case, presenting both types of results in one paper helps to understand whether a systematic approach positively influences intersectoral collaboration in the HSA.

### Conceptual framework

The specific collaborative structure involving relevant stakeholders from the education sector, health authorities, PHSs, and PSSs in HSA has been studied by Leurs et al. [13]. Inspired by the Integrated Care model, which is rooted in organizational theories [24–26] and which advocates a systematic approach to intersectoral collaboration in integrated health care [27, 28], Leurs et al. [12, 13] developed a diagnostic framework for intersectoral collaboration in the HSA, called the DIagnosis of Sustainable Collaboration (DISC) model. Using the DISC questionnaire as an assessment tool and semi-structured interviews to triangulate the results of the DISC questionnaire, Leurs et al. [29] diagnosed the collaborative structure for HSA in the southern parts of the Netherlands. Their study firstly validated the importance of the DISC factors and secondly suggested that DISC analysis can be leading in the implementation of strategies to strengthen collaboration between the sectors involved. This practice orientation makes the model very promising for the study of a systematic approach to intersectoral collaboration in CSHP.

The DISC model summarizes important factors into six main clusters that impact on the intended change and its sustainability: *collaborative support (*including the *perceptions, intentions*, and *actions* subclusters*), change management, project management, context*, *external factors* and *sustainability* (called ‘*CSHP*’ in original model). In the DISC model, stakeholders’ *collaborative support* refers to the extent to which major stakeholders agree on the problems that need to be solved and to the degree of consensus on the actions to be taken, outcomes to be achieved and boundaries to be established between the domains of various partners. In addition, it refers to stakeholders’ level of commitment to and trust in each other’s intentions to effectuate actual change (allocation of resources and manpower, adaptations to ensure sustainability, etc.) within the organization they represent (rather than acting out of self-interest only).

C*hange management* refers to the need to gradually develop a common vision of the innovation which then guides and facilitates the selection of a change perspective and change strategies, and, when necessary, the extension of the network with new actors required for the desired system change. Four perspectives for change and related change strategies have been suggested [30, 31, 27].

In the developmental and implementation phase, a *project management* structure is needed to steer the actors involved towards the accomplishment of the goals. This structure becomes self-supportive as soon as collaboration becomes formalized in the work routines of the parties involved.

The DISC model further assumes that the *context* of the stakeholders involved and several *external factors* influence the collaborative process, while especially the later factor itself is difficult to influence. The *context* supports the collaborative process when the actors involved have positive experiences with previous collaborations, have enough research power, interest and expertise in the relevant field, and recognize relevant policies which they can also influence. *External factors* include intersectoral policies, laws, and regulations at different levels (EU, local, regional, and national) relevant to HSA, encouraging attitudes of financing bodies and positive community notions and public interest in CSHP. The sixth cluster, *sustainability,* addresses the HSA itself and refers to its sustainability, with “sustainability” defined as the gradual establishment of a stable collaboration.

The relation between the DISC constructs is based on preliminary assumptions from Leurs et al.’s [29] single case study and relevant literature. Which managerial principles induce improvements on specific DISC factors has not yet been established.

Using this conceptual framework, we have studied the effectiveness and managerial processes of a systematic approach based on the DISC model in the two-year DISC-based trajectory.

## Methods

### The DISC-based trajectory

The trajectory started with DISC analyses, offering a thorough analysis of facilitating and impeding factors in five regional HSA collaborations spread across the Netherlands. The analyses showed that stakeholders’ positive perceptions about the HSA needed to be more fully translated into positive intentions and real actions and that change management and project management were hardly applied, even though they were considered crucial for this transition [19]. Based on these diagnoses, we advised the regional coordinators of the five HSA collaborations to focus on the development of effective change management and project management. In addition, and in accordance with the operationalization of the DISC model, we supported them for the period of one year in accomplishing the following steps: (1) bringing the different partners together; (2) developing a common vision; (3) identifying the possibilities and impossibilities for each collaborating partner based on the DISC analysis; (4) determining the desired collaborative structure; (5) developing a task distribution based on the information; (6) discussing the choices at the management and executive levels. Support included monthly personal/telephone contacts with the central facilitator involved in the trajectory. This facilitator was a health educator working at the PHS where the Dutch HSA and the DISC model had been developed and who was specialized in the implementation of the HSA. The facilitator provided support which basically comprised critical reflection on the collaborative process using a standard interview protocol, exchange of expertise between regions, with the facilitator as intermediary between them, and occasional peer-review of produced documents. In addition, one joint training session for regional coordinators was organized which addressed the regional coordinator’s role as process facilitators within the HSA.^1^ After support had ended, the collaborative process was monitored for an additional year using the same standard interview protocol during bi-monthly phone calls with regional coordinators. These calls were made by a researcher affiliated to Maastricht University who had also conducted the DISC analyses in the regions. The monitoring data were later used for the analysis of managerial activities induced by the systematic approach. After the one-year follow-up period, the DISC-based trajectory ended with a second round of DISC analyses which, together with the results of the first round, were used for the purpose of effect evaluation.

### Procedure and data collection instruments

Longitudinal quantitative data on the DISC factors and qualitative data on the managerial activities were collected in five of 30 (in 2008) Dutch PHS regions, from 2008 to 2011. Inclusion criterion was the ability (e.g. resources, other priorities) and willingness to start with the DISC-based trajectory at the end of 2008.

For the quantitative data collection, coordinators of the HSA working at the PHSs were asked to identify relevant stakeholders from the education sector, municipal authorities, PHSs and other PSSs. The coordinators introduced the topic of research to these persons and distributed materials for the survey (the questionnaire and a brief summary of the HSA) at the end of 2008. Data collection lasted for a total of eight weeks. Reminders were sent after an additional 4 weeks. The procedure was repeated in 2011 as a posttest.

The longitudinal quantitative data were collected using the DISC questionnaire (adapted from Leurs et al. [13]) which measures the 26 DISC factors of the 6 clusters (*collaborative support* [including the *perceptions, intentions*, and *actions* subclusters]*, change management, project management, context*, *external factors,* and *sustainability*), with 1 to 5 items each, mostly on a 5-point scale *(completely disagree: 1 to completely agree: 5)* with the additional option of ‘unknown’. Examples of items used in the questionnaire are ‘I think it is important that my organization participates in the HSA’ and ‘I think the HSA is of interest to my own organization’ (*collaborative support–perceptions*).

In the early months of 2009, the results of the quantitative pretest and the resulting recommendations (i.e. the six predefined steps) were presented to the regional coordinators in a regional report, followed by a personal meeting for further clarification between each of the regional coordinators and the facilitator and researcher. At these meetings, all regional coordinators independently indicated that priority should be given to correctly situating the HSA within their own organization and positioning it together with PSSs in the region. Consequently, managerial activities carried out during the trajectory mainly targeted the representatives of PHSs and PSSs, so only quantitative and qualitative data concerning these stakeholders are included in the current paper.

During the one-year period of support (mid 2009-mid 2010) and the one-year follow-up period (mid 2010-mid 2011), qualitative data on the collaborative process were collected using a standard interview protocol including the following questions: Which activities have been conducted for the purpose of collaboration? Which activities are planned for the purpose of collaboration? Have any documents been produced in relation to the collaboration? Are there any other developments influencing the collaborative process? Minutes were made of these interviews and sent to regional coordinators for approval. Relevant documents such as project proposals and project plans (multiyear plans and work plans) were collected. Nvivo analysis was used to extract managerial activities from the interview minutes and the collected documents, in order to open up the black box of collaborative processes in the DISC-based trajectory.

By the end of 2011, after the second round of DISC analyses, the regional coordinators received a final report with the regional results.

In accordance with Dutch regulations, no ethical approval was required for this study [32].

### Participating PHS regions

Five cases of HSA collaboration were studied, with different characteristics and starting situations. Table 1 lists these for each of the five PHS regions, in ascending order of size. We decided to study intersectoral collaboration in a variety of situations, to be able to collect more data than would have been possible in a single case study, and to enhance the generalizability of the study results.

**Table 1:** Characteristics of the five PHS regions and their CSHP at pretest

	PHS region 1	PHS region 2	PHS region 3	PHS region 4	PHS region 5
Stakeholders approached from school type	Primary education	Secondary education	Primary education	Secondary education	Secondary education
Working area (at pretest)	12 municipalities (primary education: ±324 schools)	13 municipalities (secondary education: ±12 schools on 25)	14 municipalities (primary education: ±214)	20 municipalities (secondary: ±16 schools boards)	26 municipalities (secondary education: ±30 schools)
Year of CSHP adoption	Primary and secondary: 2009	Primary education: 2006	Primary education: 2009	Primary education: 2008	Primary education: 2009
		Secondary education: 2008	Secondary education: 2008	Secondary education: 2008	Secondary education: 2009
Manpower for CSHP at PHS	Primary education: 3 health promoters	Secondary education: 4 health promoters, 2 epidemiologists	Primary education: 5 health promoters, 2 youth health care professionals	Secondary education: 4 health professionals	Secondary education: 2 health promoters, 4 youth health professionals
CSHP delivery	Primary eduaction: single service point	Primary education: newsletter	no	no	no
Health promoting school advisor in CSHP	no	Health promoting no school advisors from PHS, Youth Care, Education Support Service, Mental Health Care	no	no	no
Collaboration with PSSs in CSHP	Primary education: Education Support Service, Mental Health Youth Care, Addiction Care, Justice, Dietician, Sports company	Primary education: Organizations of health promoting school advisors, Addiction care, Welfare and YHC	no	Previous project: Mental Health Care, Justice and Welfare	no
	Meet 1 time a year	Meet six times a year			
Collaboration with municipalities in CSHP	no	Primary education: PHS informs about the healthy school approach via general PHS-journal	no	no	no
Collaboration with schols in CSHP	Primary education: PHS informs schools via single service point at PHS	Primary education: PHS recruits schools for the HSa	Primary education: PHS recruits schools for the HSA	no	no

PHS: Public Health Service; PSS: Public Service Stakeholders

### Participants

A total of 158 stakeholders at pretest and 132 potential stakeholders at posttest were approached by the regional coordinators for the DISC questionnaire. Contacted PHS professionals included health promoters, epidemiologists, pediatricians, and youth nurses. PSSs came from the domains of addiction, mental health, social welfare, and security, and other services like educational support services, dietician centers, and sports companies. The five regional coordinators who were followed and interviewed during the advice trajectory all worked at PHSs. All of them were health promoters.

### Quantitative data analyses

During data cleaning, the ‘unknown’ option was recoded to ‘1’ (negative value) as it apparently indicated the absence of a particular DISC factor. Missing values for one item in scales with four or more items were replaced by the mean of the other items.

Cronbach’s alphas were calculated for the different scales operationalizing the DISC factors. These appeared to be lower than .60 for three of the 13 multi-item scales: willingness to change, organizational characteristics, and characteristics of CSHP (Table 1)*.* The items of these scales were therefore included separately in the analyses. Descriptive statistics were used to establish the mean values for the DISC factors at both measurements.

Multi-level analyses with linear mixed regression including a random intercept were performed for the five HSA collaborations to analyze changes in staff and organizations that occurred between pretest and posttest. The random intercept model enabled us to include in the analyses both respondents with complete data and respondents who participated at pretest or posttest only. This allowed for a more reliable estimation of the general trend than an analysis based only on complete cases.

To test whether relevant measures had improved significantly at posttest, multi-level analyses (F-test) were conducted with time of measurement (pretest versus posttest) as the fixed factor and one of the DISC factors as dependent variable. We corrected for multiple testing using Holm’s method, by first ranking the p-values of the analyses by magnitude and then multiplying them by their rank number (where the largest *p*-value gets the lowest rank and the smallest *p*-value gets the highest rank). Ranking was performed for all factors within a cluster (e.g. change management). In the case of subclusters, ranking was performed for all factors within a subcluster (e.g. stakeholder’s perceptions).

In addition we calculated effect sizes based on the intercept and the error variances for each measure, in order to weight the effects. The effect sizes were interpreted according to Cohen’s [33] categorization, in which *d* = 0.20, 0.50 and 0.80 represent small, medium, and large effects, respectively.

Statistical analyses were conducted using SPSS 20.

### Qualitative data analyses

Document analysis included 65 min of the standard protocol interviews with the regional coordinators, 39 of which were held by the facilitator and 26 by the researcher, and the project proposals and project plans regarding the HSA from each region. Document analysis with Nvivo 9 was independently performed by two researchers, the researcher who conducted the DISC analyses and a research assistant with expertise on the topic. Ambiguities of coding were resolved by discussion. Data from documents were mainly used to verify regional coordinators’ self-reports on managerial activities. Initial coding focused on the identification of managerial activities. Several iterations of analyses resulted in a classification scheme of managerial activities.

We triangulated our data by studying information from various types of sources (qualitative and quantitative data) and by having the coding of the qualitative data done by two independent researchers [34].

## Results

### Quantitative results

#### Response

Of the total of 158 stakeholders who were approached at pretest and 132 at posttest, 90 (57 %) and 69 (52 %), respectively, were included in the analyses. There were various reasons for dropping out. Organizational tasks of collaborating parties changed in response to mergers, reorganizations, government cutbacks and changing policies. Also, collaborative goals became more specific as collaboration progressed, which led to a poorer fit between the agenda of some organizations and the collaborative agenda.

Table 2 indicates that the regional coordinators had set slightly different priorities within the collaboration, since the numbers of representatives from the two sectors who were approached varied between regions.

**Table 2:** Response of stakeholders

	PHS region 1	PHS region 2	PHS region 3	PHS region 4	PHS region 5	Total
Pretest (responded/approached)	PHS: 5/5	PHS: 9/9	PHS: 25/32	PHS: 11/19	PHS: 20/64	PHS: 70/129 (54 %)
	PSS: 5/7	PSS: 5/6	PSS: −	PSS: 10/16	PSS: −	PSS: 20/29 (69 %)
	Total: 10/12	Total: 14/15	Total: 25/32	Total: 21/35	Total: 20/64	90/158 (57 %)
Posttest (responded/approached)	PHS: 6/9	PHS: 7/7	PHS: 20/36	PHS 8/8	PHS: 10/48	PHS: 51/108 (47 %)
	PSS: 5/7	PSS: 3/3	PSS: −	PSS: 9/12	PSS: 1/2	PSS: 18/24 (75 %)
	Total: 11/16	Total: 10/10	Total: 20/36	Total: 17/20	Total: 11/50	69/132 (52 %)

PHS: public health service; PSS: public service stakeholders

#### What are the pretest-posttest differences regarding the DISC factors?

Our results indicate significant differences between pretest and posttest in all six DISC categories (Table 3).

**Table 3:** Description of DISC constructs (adapted from Leurs, Mur-Veeman et al. (2008)), reliability, response and linear mixed model regression analysis

Description of DISC clusters and DISC subclusters	DISC factors	Cronbach’s alpha		Pretest		Posttest		Linear mixed model of regression analysis				Effect size
	(based on single-item or multi-item scales)	Number of items		Means		Means						
		(N)	alpha	N	Mean (SD)	N	Mean (SD)	b	SE (b)	F	corrected *p*-value^a^	Cohen’s d
**Collaborative support**												
The collaborative support can be assesed at the levels of perceptions, intentions and actions of the parties involved.												
-Perceptions												
Intersectoral collaboration envolves more smoothly when participating organizations share goals and interests, perceive positive outcomes supportive of their own goals, are able to reach consensus on the goal of the collaborative parties are involved in the collaborative process.	Goals	5	.876	90	4.17 (.77)	67	4.22 (.66)	-.021	.091	.051	1,644	0.03
	Importance	3	.721	88	3.67 (.62)	65	3.73 (.75)	.023	.106	.046	0,83	0.03
	Win-win	1		89	3.75 (.84)	66	3.73 (.94)	-.052	.141	.134	2.253	0.06
	Ideological consensus	4	.859	90	2.92 (1.23)	67	3.60 (1.06)	.582	.171	11.649	.006**	0.50
	Domain consensus	1		90	2.44 (1.26)	67	2.96 (1.19)	.482	.191	6.356	.065	0.39
	Involvement	2	.662	87	2.25 (1.14)	67	2.64 (1.11)	.389	.180	4.696	.128	0.34
-Intentions												
Parties involved should start with the intention to trust each other (if not present, this needs to be worked on first), the intention to commit themselves to the collaborative process and its subject and the intention to make changes within their own orgnization, if needed, in favor of the collaborative process.	Willingness to trust	1		90	2.74 (1.54)	67	3.03 (1.50)	.199	.224	.795	.0375	0.13
	Willingness to commit	1		90	2.78 (1.36)	67	3.42 (1.20)	.633	.204	9.677	.008**	0.49
	Willingness to change	2	.530									
	-Available room for change			90	2.62 (1.40)	67	2.82 (1.28)	.152	.205	.550	0.92	0.11
	-Things have to change			90	1.94 (1.10)	67	2.27 (1.07)	.324	.175	3.440	.198	0.30
-Actions												
The collaborative process nay induce a wide variety of actions, ranging from the implementation of major innovations within one’s own organizations to the inclusion of relatively minor adaptations of regular procedures. The actions may involve a reallocation of resources as well. Whatever actions results from a collaborative process, it is important that these are formalized in order to enhance sustainability. The level of formalization needed depends mainly on the type of action itself.	Changes	1		90	2.69 (1.55)	67	3.08 (1.33)	.459	.222	4.258	.084	0.31
	Resources	2	.660	90	3.41 (1.28)	66	3.68 (1.09)	.289	.193	2.247	.136	0.24
	Formalization	1		90	2.68 (1.29)	67	3.31 (1.13)	.657	.182	13.074	.003**	0.53
**Change management**												
The aspired change requires management by one or a small group of leaders. Establishing a succesful collaboration requires individual and collective leadership skills to guide the developmental process. Change management strategies should fit the chosen innovation perspective, and be supportive of the health promotion subject. The most relevant actors are included and where missing, this is accomplished by extending the network of the leaders of the collaborative process.	Vision	3	.866	89	3.40 (1.15)	67	3.91 (1.02)	.495	.154	10.359	.008**	0.45
	Innovation perspective	4	.694	89	2.55 (.97)	67	3.05 (.91)	.430	139	9.534	.009**	0.45
	Change strategy	4	.714	89	2.36 (.99)	67	2.84 (.94)	.433	147	8.688	.008**	0.44
	Network development	1		89	2.43 (1.40)	67	2.39 (1.11)	-.039	208	.035	.852	0.03
**Project management**												
During the development and initial implementation phase, the collaborative process is dealt with as a project in a project management structure. This includes deciding who are the actors in the project, what they need to do and how they operate (planing procedures, evaluation, communications, etc.). This project management structure fades out when the subject of the collaborative process is being integrated (or close to being integrated) in regular work and the alliance becomes sel-supportive.	Actors. Task.	3	.860	89	2.44 (1.26)	66	2.97 (1.10)	.507	.180	7.962	.006**	0.44
	Communicaton.											
	-Actors			89	2.43 (1.41)	67	3.04 (1.38)	.621	.221	7.887	.006**	0.44
	-Tasks			90	2.44 (1.26)	67	2.96 (1.19)	.482	.191	6.356	.026*	0.39
	-Communication			90	2.46 (1.33)	67	2.86 (1.36)	.351	.205	2.919	.180	0.26
**Context**												
The collaborative process evolves in a context which can be influenced by the partners themselves. When parties have had positive experiences with each other in previous collaborative processes, need less energy for internal changes, have more research power and feel more supported by policies which they can influence as well, they will be more open to sustainable collaborative processes supporting intersectoral health promotion.	Organizational characterisctic	3	.489									
	-Open to innivations			89	3.80 (1.04)	67	3.96 (.96)	.160	.158	1.020	.942	0.16
	-Organizational problems			89	2.78 (1.32)	67	1.94 (1.03)	-.788	.188	17.609	.00**	−0.66
	-Positive experience with previous collaboration^c^			89	3.16 (1.45)	67	3.61 (1.35)	.455	.228	3.980	.192	0.32
	Research power	1		89	3.38 (1.20)	67	3.46 (1.20)	.092	.194	.194	.636	0.32
	Relevant policies	1		89	3.15 (1.35)	67	3.33 (1.20)	.182	.208	.208	.636	0.14
**External factors**												
The collaborative process is influenced by a number of factors that are beyond the control or influence of the alliance itself.												
Clear, preferably intersectoral policies, laws and regulations providing challenging and sound goals for health promotion, may enhance the collaborative process.	Policy and regulations	2	.600	90	3.29 (1.08)	67	3.83 (.90)	.552	.154	12,922	.000***	0.55
Limiting factors may be poorly defined boundaries between policy domains, contradictory policies of different public sectors and policies focusing on the transformation of public organizations into private.												
An encouraging and accomodating attitude on the part of financing bodies and commitment to provide the necessary funding over a longer period to prevent brain drain during the initial developmental phase supports the collaborative process.	Attitude of financing organizations	1		89	2.64 (1.53)	67	2.73 (1.55)	.091	.250	.133	.716	0.04
Community notion can be regarded as an added value for the individual interests of each party and can additionally stimulate organizations to work together on coordinated school health promotion. Incentives, policies and regulations can increase community notion for coordinated school health promotion, as can parents, school staff and collaborating parties who show social interest in coordinated school health promotion.	Community notion	1		90	2.50 (1.38)	67	3.00 (1.37)	.362	.211	2.938	1.80	0.26
**Sustainability**												
The colloborative process influences the development of the coordinated (school) health promotion and supports the move towards sustainability (goal): Under the continuous influence of the collaborative process an idea is elaborated and is formalized into regular working routine. During this process the subject of the collaborative process evolves: it ‘changes colour’ under the influence of the collaborative process itself.	Characteristics of HSA	6	.548									
	-Project vs. regular work			84	2.14 (1.16)	65	2.55 (1.26)	.429	.183	5.256	.072	.36
	-Network support vs. individual actions			82	2.91 (1.20)	66	3.20 (.98)	.282	.135	2.380	.125	.26
	-Research vs. practical			83	3.37 (.95)	64	3.80 (.78)	.522	.161	15.065	.000***	.59
	-systematic vs. ad-hoc			81	3.38 (1.06)	66	3.74 (.93)	.389	.136	5.816	.010*	.39
	-Practical vs.theoretical			82	3.20 (.90)	66	3.41 (.80)	.212	.166	2.422	.020*	.25
	Single service points vs. fragmentation			80	2.85 (1.11)	66	3.29 (.97)	.487	.089	8.525	.030*	.46

^a^p-values corrected according to Holm procedure: *p ≤.05, **p <.01, ***p <.001

##### Collaborative support

Significant differences between pretest and posttest were found, with medium effect size. At posttest, the parties involved had more shared views about ways to realize the HSA (*perception of ideological consensu*s), had greater intentions to commit to the collaborative goal (*intention to commit)* and reported more formalization of the HSA within their organizational policies (*action of formalization)*.

##### Change management

Change management improved significantly, with gains of almost medium effect size, specifically regarding the presence of a clear and systematically developed vision, the innovative perspectives pursued and the change strategies employed.

##### Project management

Respondents evaluated the project management more favorably at posttest than at pretest. Gains were small, though almost of medium magnitude. Further exploration of project management showed that it was especially the awareness among stakeholders involved and their responsibilities which had increased.

##### External factors

Significant improvements of medium size were found for the external context. According to the respondents, the HSA appeared to fit in with school health policies and public health policies (*policies and regulations*).

##### Context

The internal context for the implementation of the HSA, however, had deteriorated at posttest. The number of problems within their own organization increased, an effect of medium magnitude.

##### Sustainability

Finally, respondents evaluated CSHP significantly better at posttest than at pretest. At posttest, they perceived the HSA more as practical and less as research (ES >0.50) or as theory (ES <0.20). Furthermore, the HSA was evaluated more as permanent and less as ad-hoc (ES <0.20). In addition, respondents perceived less fragmentation of school health promotion and were more aware of the existence of a single service point at PHSs for health promotion programs and expertise. This effect was almost of medium effect.

### Qualitative results

#### How was the DISC-based trajectory operationalized by the regional coordinators?

**Fig. 1:**
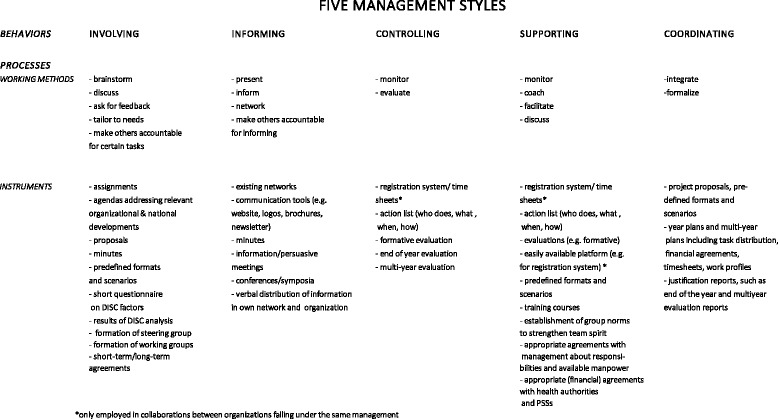
Operationalization of the DISC advice by regional coordinators: five management styles

We identified a great variety of working methods and instruments that the regional coordinators applied in response to the DISC-based advice, which clustered into five main management styles (Fig. 1). Below we summarize how these management styles were used in practice.

##### Involving

Regional coordinators created spaces for negotiation between stakeholders about a common vision, a possible collaborative structure and a suitable division of tasks. They used different working methods and instruments to induce people to think about these aspects and to give everybody a voice in the decision-making process. They involved stakeholders through discussions and brainstorm sessions, and encouraged their involvement by means of prepared agendas, short questionnaires, DISC results or assignments. An example of an assignment used for the systematic development of a common vision is shown in Table 4. Based on the results of the individual assignments that had been completed by each stakeholder, the regional coordinators came up with a proposal for a common vision. Comparable assignments were used to compare financing systems and policy cycles between collaborating parties.

**Table 4:** Work assignment: creating a common vision

1. Describe the mission of your organization/department towards health in education.
2. Describe the added value of the HSA for your own organization/department.
3. Describe the contribution your organization/department can provide to the HSA.
4. Describe the preconditions for your organization/department for any contribution to the HSA.

Additionally, they stimulated active participation by making collaborating partners responsible for certain tasks through the formation of steering groups and working groups. They also created moments for feedback on minutes, proposals, predefined formats/scenarios, and plans, to create a common perspective and commitment, and they tailored agreements to the possibilities and impossibilities of collaborating parties to facilitate alignment of policies. For example, when stakeholders were unable to commit to long-term agreements, then short-term agreements about investments and task distributions were made instead.

##### Informing

Regional coordinators shared plans with distal stakeholders (i.e. municipal authorities, PSS managers), raised awareness of HSA among ‘potential’ stakeholders and provided everybody who worked with the HSA (distal and proximal stakeholders) with relevant updates and tools. For example, regional coordinators informed stakeholders at local and regional levels through newsletters and websites. In addition, they networked with relevant informants during conferences and symposia. They held presentations during persuasive and information meetings for potential partners and municipalities. They also assigned stakeholders to be responsible for the distribution of information in their organization and their networks. Finally, they used minutes of meetings as a source of commitment and progress tracking for closely involved stakeholders.

##### Controlling

Planning and control systems were set up to inform the collaboration about whether the plans that had been made would bring about the desired effects, whether the resources allocated (e.g. staff, time) were realistic, but also to identify deviations at operational level and to intervene in time. The regional coordinators employed different systems at different levels. For example, at operational level, they used available registration systems to monitor and evaluate activities (e.g. the number of schools visits performed by the health promoting school advisors, the time they dedicated to school visits) and worked with action lists which they used as checklists during meetings. At tactical level, the coordinators used different types of evaluations (e.g. end-of-year evaluations) to check whether the intended outcomes and budgets were met.

##### Supporting

The coordinators organized different types of support. At operational level, they used monitoring in combination with active coaching. For the monitoring part, they used information from the registration system, from action lists and different types of evaluations. For active coaching, they used tools from the 2010 practitioner guide [7], including predefined formats and scenarios for discussions with particular stakeholders, and provided these on a platform that was easily accessible to health professionals. The regional coordinators also encouraged participation in training courses and discussion of group norms to strengthen the team spirit (e.g. supporting each other in the attainment of the goals established). At local level, regional coordinators facilitated demand-driven practices among collaborating parties by establishing appropriate financial and other arrangements with municipalities (and possibly PSSs). At managerial level, the regional coordinators created clearly defined agreements about responsibilities and manpower, to prevent resistance and fears among the representatives involved.

More active forms of control and supportive behaviors were used in collaborations between organizations that fall under one umbrella organization, with clear agreements on resources and manpower and one management (e.g. youth health care and public health officials working at PHSs).

##### Coordinating

Coordinating activities were central to all other management activities. Coordination included an interactive, integrative process of collecting information, interpreting it, determining knowledge requirements, outlining the next steps and elaborating ways in which results could best be presented to the parties involved (e.g. proposals, predefined formats), followed by formalization of final decisions (e.g. multi-year plans).

## Discussion

The current study had two aims: first to investigate the effectiveness of a systematic approach to the development of intersectoral collaboration in CSHP and second to open up the ‘black box’ of the collaborative processes involved. These aspects were investigated using the data from a two-year DISC-based trajectory. We examined longitudinal quantitative data from the trajectory to find out whether collaboration had improved in terms of the DISC factors after participation in the DISC-based trajectory (research question 1), and studied the longitudinal qualitative data to assess whether managerial activities were employed by regional coordinators in response to the trajectory, and if so which ones (research question 2). Presenting both results, quantitative and qualitative, in one paper provides additional understanding of the origins of the observed effects.

As regards our first research question, the quantitative data showed remarkable improvement (of almost medium effect size) in change management and project management. In addition, gains of medium magnitude were found on the three subclusters of *collaborative support* (i.e. *perceived consensus, intention to commit,* and *formalization*), as well as on one measure of *external factors* (i.e. *perceived alignment between policies*), and on four measures of *sustainability* (i.e. *theoretical vs. practical, research vs. practical, ad-hoc vs. systematic, fragmentation vs. single service point*). These improvements were found despite an increase in organizational problems reported by stakeholders involved (due to e.g. mergers, reorganization, financial cutbacks). As regards our second research question, our qualitative data showed that regional coordinators undertook different activities in response to the DISC-based trajectory, activities which clustered into five managerial styles and basically addressed (1) *involving* stakeholders in the decision-making process, (2) *informing* them about decisions and progress made, (3) *controlling* and (4) *supporting* their task accomplishment, (5) and *coordinating* the collaborative process.

The improvements that we found in terms of *change management, project management*, and *collaborative support* seem to be the result of having encouraged regional coordinators to identify common grounds with the stakeholders regarding several aspects. These aspects included the establishment of a common vision, a common collaborative structure, and a suitable task distribution, while considering stakeholders’ individual interests. In this respect our qualitative data provide some indications that regional coordinators have created the necessary opportunities for stakeholders to freely voice their suggestions, doubts, and wishes regarding these aspects (the *involving* management style). Similar findings have been reported by others, who found that creating spaces for negotiation (e.g. brainstorming, discussion) can enhance the consensus on collaborative goals and necessary actions, and thereby promote the formulation of commitments in collaborations [35, 20, 36].

The same reasoning seems to apply to the increased degree of formalization we have found at posttest. We advised regional coordinators to discuss choices at the management and executive levels. The qualitative data indeed indicate that regions consolidated agreements in documents at different levels (the *coordinating* management style). Bohlmeijer et al. [35] reported that written documents can be regarded as the visible results of the negotiations and are an important indicator of formalization. Koelen et al. [37] showed that such formalization builds employees’ accountability, which acts as an important driving force for action in Dutch health organizations.

As an adverse development, we have observed that the internal context of the parties involved deteriorated. It is conceivable that this result is related to the internal developments (e.g. mergers, reorganizations) that the organizations were going through during the trajectory, in response to government cutbacks due to the financial crisis in the Netherlands. In addition, it could be the consequence of the new four-year public health policy cycle which organizations had entered in 2011 and which obliged them to translate new national health promotion objectives into concrete strategies to improve health at local level. We observed that these dynamics often forced collaborating parties to make choices which were not in interest of the collaboration. Time and staff were mainly dedicated to the reorientation. Legislation changed, as did organizational tasks. Some PSSs left the collaboration, others reduced their investments. The reconciliation of collective and individual interests has been reported by others as a recurring dilemma in collaborations. While stakeholders achieve collaborative goals, they also have to fulfil the individual organization’s mission and respond to organizational developments and problems [20, 21, 37–42]. This finding may explain the state of the collaboration after three years, where collaboration shows no improvements in terms of collaborative actions as yet. Even if agreements were reached, this seemed to require much more time.

Next to the effort to reconcile individual and collective interests, our qualitative and quantitative data strongly suggest that regional coordinators recognized relevant policies for intersectoral collaboration and tried to influence them to promote collaboration. Regional coordinators used the ‘windows of opportunity’ [43] that emerged from the dynamic context for this purpose. They placed the HSA and the collaborative structure on the agenda of meaningful internal and external meetings (the *informing* management style). They made connections and opportunities visible to relevant actors (the *coordinating* management style). They developed supporting policies and financial agreements between municipal authorities and PHSs (the *supporting* management style). Our quantitative data confirm greater alignment between health policies, school policies, and the HSA after the trajectory (*external factors*). De Leeuw [27] reported comparable skills by ‘social entrepreneurs’ who were able to influence policy agendas. These social entrepreneurs had the abilities to obtain an overall view of the various perspectives of stakeholders, to broker commitments of stakeholders into networks and to reflect on their own position and that of the stakeholders. They influenced the policy agenda by bringing problems, solutions, and the right stakeholders together.

By contrast, our study shows that directive behaviors, which focus on task performance, such as planning and control systems (the *controlling* management style) were less commonly employed, and when they were used, it was mostly in organizations that fall under one umbrella organization. This probably indicates the difficulty of employing this type of behavior in collaborations lacking formal authority [35, 41].

Finally, it is encouraging to find that stakeholders expressed more favorable judgments about the sustainability of the collaboration at posttest (i.e. more systematic, more practical, more demand-driven practices). Although the absence of comparable studies makes it difficult to compare our findings, there are studies that support the view that a systematic, step-wise approach to change can give direction and transparency [35, 44–46]. A study involving the provision of professional support for school staff to implement the CSHP acknowledged that professional support can enhance the acquisition of organizational knowledge and its translation into practice [47]. Lastly, the finding that school health promotion developed from fragmentation towards a single service point for health promotion (i.e. demand-driven practices) provides additional evidence that collaborative efforts start to pay off and contribute to collaborative goals.

Based on the above interpretations, it is plausible to postulate that regional coordinators employed managerial activities in response to the DISC-based trajectory, which have contributed to the observed improvements in terms of the DISC factors. In this respect the combination of the qualitative and quantitative data was an important strength of our study. It allowed data triangulation and a combined study of effects and processes. This in turn provided important insights into the causes of the observed effects. This strength partly offsets the weakness of the quantitative results based on a pretest-posttest design, which as such limited the opportunities to draw causal inferences due to possible history and maturation biases and Hawthorne effects [48]. In addition, data source and investigator triangulation, as well as the member check of analyzed minutes contributed to the objectivity (i.e. confirmability) and credibility of our findings. Furthermore, studying multiple cases gave us the opportunity to collect qualitative data from various cases, which differed in several characteristics and staring situations, and thereby increased the transferability of our conclusions. Finally, we tried to enhance readers’ judgments about the dependability of qualitative findings through a thorough description of the inquiry process and interpretation of findings against the context of the studied collaboration [49, 50]. Nevertheless, some limitations to our study should be considered. Despite being based on multiple cases, our quantitative research suffered from a small sample size, due to the small number of stakeholders involved. In addition, drop-out affected our sample size, though we minimized its detrimental effects by applying suitable analysis techniques (i.e. including all available cases in the analysis). Furthermore, selection bias may have affected our data because of the voluntary participation of PHS regions in the DISC-based trajectory and because the stakeholders were not randomly selected. In addition, regional coordinators decided to give priority to a particular sector in the collaboration. The desire to achieve their own organizational goals (e.g. youth health promotion) may have led their choices. Finally, the data gathered for the purpose of support may not necessarily capture all managerial activities that the regional coordinators employed, so our overview might not be exhaustive and might need further elaboration. In addition, the stage of the collaboration limited the number of managerial activities that could be studied, activities which can help manage the transition from formalization to collaborative action. These activities will thus need special attention in follow-up studies.

## Conclusions

To conclude, our conclusion study provides preliminary evidence for the effectiveness of a systematic approach to intersectoral collaboration based on the DISC model. In addition, it offers important insights into the black box of managerial processes related to this kind of approach.

Our findings suggest that when collaboration is to be established in a systematic way, a thorough analysis of facilitating and impeding factors can help to formulate an appropriate strategy. In addition, it seems to indicate that each step of this strategy should provide enough space for negotiation and be concluded with formalized agreements, so that employees are made accountable for collaborative actions. Support for this process can probably be provided by partnership managers who can devise creative working methods and instruments to manage the collaboration, and who have the skills to function as social entrepreneurs in dynamic contexts. The byproducts of our study are a strategy to improve the change and project management in intersectoral collaboration and a large repertoire of managerial working methods and instruments. Both might inspire health professionals engaged in comparable partnership endeavors. Assistance on demand might be necessary to support them in the acquisition, assimilation, and application of the new knowledge.

## Abbreviations

DISC model: Diagnosis of sustainable collaboration model
CSHP: Comprehensive school health promotion
HSA: Healthy school approach
PHSs: Public health services
PSSs: Public service stakeholders

## Footnotes

1 The training was based on a national supportive manual for health professionals involved in the HSA (RIVM Centrum Gezond Leven [7] and co-organized by the Netherlands School of Public and Occupation Health.
